# Prevalence and treatment of gout among patients with chronic kidney disease in the Irish health system: A national study

**DOI:** 10.1371/journal.pone.0210487

**Published:** 2019-01-25

**Authors:** Elshaeima Mohammed, Leonard D. Browne, Arun Kumar A. U., Fahd Adeeb, Alexander D. Fraser, Austin G. Stack

**Affiliations:** 1 Division of Nephrology, Department of Medicine, University Hospital Limerick, Limerick, Ireland; 2 Graduate Entry Medical School, University of Limerick, Limerick, Ireland; 3 Department of Rheumatology, University Hospital Limerick, Limerick, Ireland; 4 Health Research Institute, University of Limerick, Limerick, Ireland; University of Colorado Denver School of Medicine, UNITED STATES

## Abstract

**Background:**

Gout is a common inflammatory arthritis associated with adverse clinical outcomes. Under treatment is common in the general population. The aim of this study was to determine the prevalence of gout and its treatment among patients with chronic kidney disease (CKD).

**Methods:**

We conducted a multi-centre cross sectional study of patients (n = 522) who attended specialist nephrology clinics in Ireland. Standardized data collection tool recorded clinical characteristics and medication use at clinic visits and kidney function was assessed with standardised creatinine measurements and Estimated Glomerular Filtration Rate (eGFR). The prevalence of gout and the corresponding use of urate lowering therapies (ULT) were determined. Multivariate logistic regression explored correlates of gout expressed as Odds Ratios (OR) and 95% Confidence Intervals (CI) adjusting for demographic and clinical characteristics.

**Results:**

Overall prevalence of gout was 16.6% and increased significantly from 7.5% in Stage 1–2 CKD to 22.8% in stage 4–5 CKD, *P*< 0.005. Prevalence increased with age (P < 0.005) and was higher in men than women (19.1% versus 10.3% P< 0.005). Overall, 67.9% of gout patients with CKD were treated with ULT, and the percentage increased with advancing stage of CKD from 55.6% in Stage 1–2 to 77.4% in Stage 4–5, P<0.005. Multivariable modelling identified men (vs women), OR, 1.95 (0.95–4.03), serum albumin, OR 1.09 (1.02–1.16) per 1 g/L lower, poorer kidney function, OR 1.11 (1.01–1.22) per 5 ml/min/1.73m^2^ lower, and rising parathyroid hormone levels, OR 1.38 (1.08–1.77) per 50 pg/ml higher as disease correlates.

**Conclusions:**

Gout is common in CKD and increases with worsening kidney function in the Irish health system. Over two thirds of patients with gout were receiving ULT, increasing to 77% of patients with advanced CKD. Greater awareness of gout in CKD, its treatment and the effectiveness of treatment strategies should be vigorously monitored to improve patient outcomes.

## Introduction

Gout is a common inflammatory arthropathy caused by the deposition of monosodium urate crystals in joints and soft tissues. In the general population, the prevalence of gout varies worldwide from 0.1% to approximately 10% and incidence rates vary from 0.3 to 6 cases per 1,000 person-years [1]. In addition to causing excruciating arthritic pain, gout is associated with premature death, classically explained by a high frequency of comorbid conditions, especially renal and cardiovascular diseases [2–4]. Gout is associated with a progressive functional impairment, reduced quality of life, lost productivity and increased mortality [5,6]. Recent observational studies implicate both hyperuricaemia and gout as possible risk factors for progression of chronic kidney disease (CKD) suggesting that the treatment of these conditions may lead to measurable clinical benefits [7,8]. Several small clinical trials have found that treatment with Urate-Lowering Therapy (ULT) reduced the progression of kidney disease [9,10]. Furthermore, a recent meta-analysis of clinical trials with over 1, 200 patients found that treatment with ULT significantly reduced the risk of major renal and cardiovascular events [11]. This emerging evidence would suggest that treatment and control of gout is especially important among patients with impaired kidney function.

Few studies have examined the burden of gout among individuals with impaired kidney function in the general population or with CKD within the health system. A report from Krishnan using data from the 2009–2010 National Health and Nutrition Examination Survey (NHANES) in the US found that the prevalence of self-reported gout increased from 2.9% in patients with normal renal function to 33.3% among those with glomerular filtration rate (eGFR) < 30 ml/min/1.73m^2^. Adjusting for confounding, individuals with severe renal impairment had a 6-fold higher prevalence of gout compared to those with normal kidney function [12]. Data from the German Chronic Kidney Disease (GCKD) cohort, a prospective observational study of 5,085 patients, found a prevalence of 24.3% among patients with pre-existing CKD which increased to 35.6% among those with GFR < 30 ml/min/1.73m^2^ [13]. These studies would suggest that gout is highly prevalent among patients with pre-existing CKD and may contribute significantly to morbidity from arthropathy and accelerated kidney disease progression. Despite these studies there are several unanswered questions with regard to the burden of gout and its management among patients with CKD in the health system. For example, it is unclear to what extent patients with gout who attend specialist renal clinics are treated with ULT and whether treatment rates vary across stage of CKD.

Given the paucity of data on the burden and management of gout in health systems we conducted a multicentre cross–sectional study to determine the prevalence of gout and concurrent treatment strategies among CKD patients within the Irish health system.

## Materials and methods

### Study design

This study was a multicentre cross-sectional study of adult patients with CKD treated at 18 adult specialist nephrology clinics during the first 2 weeks of December 2012 and 2013. All nephrology clinics were invited to participate in the audit and a consecutive sampling approach for patient selection was adopted. The clinics were geographically dispersed across six health regions in the Republic of Ireland (West, Midwest, Northwest, Midlands, East and Southeast). Patients less than 18 years of age or receiving dialysis were excluded. A standardised data collection tool was used to capture anonymised clinical information from medical case records, laboratory information systems and physician clinic letters. Demographic and clinical characteristics were captured including primary cause of kidney disease, comorbid medical conditions, prescribed medications and laboratory values recorded within the previous 3 months [or within 6 months for specific laboratory values for iron indices, parathyroid hormone (PTH), lipids and haemoglobin A1c (HbA1c)]. The study was approved by the Ethics Committee of University Hospital Limerick.

### Definition of gout

Gout was defined as the presence of a documented clinical diagnosis of gout on medical case records and/or physician clinic letters or if the patient was receiving ULT. The Estimated Glomerular Filtration Rate (eGFR) was calculated using the Chronic Kidney Disease Epidemiology Collaboration (CKD-EPI) equation [14] and CKD was classified according to the Kidney Disease Improving Global Outcomes (KDIGO) guidelines [15]. The following eGFR categories were defined: ≥ 60 ml/min/1.73 m^2^ (Stages 1–2), 30–59 (Stage 3) and <30 (Stages 4–5).

### Statistical methods

Descriptive statistics were calculated for continuous variables (reported as mean values and standard deviations or median and IQR where appropriate) and categorical variables (reported as numbers and percentages). Comparisons across groups were made using Fisher’s exact tests for categorical variables and Kruskal-Wallis test for continuous variables. Multivariable logistic regression models were fitted to explore the associations of demographic and clinical factors with prevalent gout. Sequential age, and age and sex adjusted models were developed to examine relationships. The explanatory variables included age modelled as 5-year intervals, sex, comorbid medical conditions, laboratory values recorded prior to clinic visit, and prescribed medications. A final multivariate model was constructed to identify the relative contributions of demographic, clinical and treatment factors with the presence of gout. Model performance was assessed using the c-statistic and the adequacy of the logistic models was tested using the Hosmer and Lemeshow goodness-of-fit test. Associations were expressed as odds ratios (ORs) and 95% confidence intervals (CIs) and all analyses were performed using R statistical software [16].

## Results

### Baseline characteristics of the study population

Table 1 outlines the basic characteristics of the study population by CKD stage. The majority were men (55.2%), white Irish (95%) and the average age was 58.2 (SD 16.9). The average eGFR was 48.4 (SD 27.7) ml/min/1.73 m^2^. The principal causes of CKD were hypertension (26.8%), glomerulonephritis (18.1%) and diabetes (12%), although for a large proportion the primary cause was classified as unknown in 13.6%.

**Table 1:** Baseline characteristics of the study population by stage of CKD.

Variable	n	Overall Cohort (n = 522)	Stage 1–2 (n = 142)	Stage 3 (n = 176)	Stage 4–5 (n = 139)	*P*-value
**Demographic**						
Age mean (SD)	515	58.2 (16.9)	48.8(14.5)	60.3(15.1)	66.3(15.7)	<0.001
Sex						
Men (%)	283	(55.2)	78.0 (54.9)	106.0 (60.2)	77.0 (55.4)	
Women (%)	230	(44.8)	64.0 (45.1)	70.0 (39.8)	62.0 (44.6)	0.568
**Race (%)**						
White Irish	479	(95.0)	126.0 (91.3)	168.0 (96.6)	132.0 (96.4)	0.051
White Irish traveller	2	(0.4)	1.0 (0.7)	0.0 (0.0)	0.0 (0.0)	0.054
White other	12	(2.4)	2.0 (1.4)	5.0 (2.9)	4.0 (2.9)	0.054
Asian	2	(0.4)	1.0 (0.7)	1.0 (0.6)	0.0 (0.0)	0.051
Other	9	(1.8)	8.0 (5.8)	0.0 (0.0)	1.0 (0.7)	0.052
**Cause of CKD (%)**						
Hypertension	138	(26.8)	31.0 (21.8)	44.0 (25.0)	48.0 (34.5)	0.045
Diabetes	62	(12.0)	12.0 (8.5)	25.0 (14.2)	23.0 (16.5)	0.105
Glomerulonephritis	93	(18.1)	35.0 (24.6)	26.0 (14.8)	21.0 (15.1)	0.052
Autosomal dominant PKD	33	(6.4)	7.0 (4.9)	16.0 (9.1)	7.0 (5.0)	0.253
Hereditary nephritis	13	(2.5)	5.0 (3.5)	6.0 (3.4)	1.0 (0.7)	0.257
Other cause of CKD	168	(32.6)	53.0 (37.3)	58.0 (33.0)	43.0 (30.9)	0.496
Not known	70	(13.6)	16.0 (11.3)	18.0 (10.2)	23.0 (16.5)	0.231
**Biopsy (%)**						
Native Kidney biopsy	75	(14.4)	27.0 (19.0)	26.0 (14.8)	18.0 (12.9)	0.363
**Comorbid Conditions (%)**						
Gout	78	(16.6)	9.0 (7.5)	29.0 (17.8)	31.0 (22.8)	0.003
Diabetes	106	(22.6)	21.0 (17.6)	38.0 (23.2)	38.0 (27.9)	0.148
Hypertension	370	(78.7)	87.0 (73.1)	131.0 (79.9)	114.0 (83.8)	0.108
Cancer	33	(7.0)	9.0 (7.6)	13.0 (7.9)	9.0 (6.6)	0.919
Heart failure	22	(4.7)	2.0 (1.7)	7.0 (4.3)	10.0 (7.4)	0.105
Thyroid disease	48	(10.2)	7.0 (5.9)	19.0 (11.6)	18.0 (13.2)	0.124
Stroke or Transient ischaemic attack	22	(4.7)	4.0 (3.4)	7.0 (4.3)	9.0 (6.6)	0.483
Chronic obstructive airways disease	22	(4.7)	4.0 (3.4)	7.0 (4.3)	8.0 (5.9)	0.607
Peripheral vascular disease	38	(8.1)	4.0 (3.4)	17.0 (10.4)	15.0 (11.0)	0.042
Coronary heart disease	75	(16.0)	11.0 (9.2)	26.0 (16.0)	33.0 (24.3)	0.005
Obesity	28	(6.0)	5.0 (4.2)	8.0 (4.9)	12.0 (8.8)	0.262
Hypercholesterolemia	127	(27.1)	28.0 (23.3)	48.0 (29.4)	43.0 (32.1)	0.287
Depression	20	(4.3)	6.0 (5.0)	6.0 (3.7)	7.0 (5.1)	0.791
Arthritis	27	(5.7)	6.0 (5.0)	11.0 (6.7)	6.0 (4.4)	0.689
Osteoporosis	30	(6.4)	7.0 (5.9)	16.0 (9.8)	4.0 (2.9)	0.053
Current or ex-smoker	52	(11.1)	15.0 (12.6)	20.0 (12.2)	12.0 (8.8)	0.583
**Physical Measurements**						
Weight (kg)	397	80.7 (17.5)	79.8 (15.9)	79.8 (17.6)	82.2 (19.5)	0.748
Pulse (beats/min)	293	75.2 (16.3)	75.6 (13.1)	73.6 (13.6)	75.7 (19.3)	0.473
Systolic BP (mmHg)	481	137.7 (19.6)	131.8 (16.6)	138.5 (19.0)	141.7 (21.7)	<0.001
Diastolic BP (mmHg)	481	77.7 (13.3)	78.7 (13.8)	77.7 (12.9)	76.7 (12.3)	0.302
**Urine tests**						
Protein: creatinine ratio	136	136.0 (246.8)	49.0 (71.3)	150.1 (348.0)	209.7 (226.3)	<0.001
Albumin: creatinine ratio	51	101.9 (214.9)	22.7 (28.1)	93.4 (239.2)	208.2 (280.0)	0.004
**Prescribed Medications (%)**						
ACE-I	114	(21.8)	37.0 (26.1)	45.0 (25.6)	21.0 (15.1)	0.040
ARB	93	(17.8)	27.0 (19.0)	34.0 (19.3)	26.0 (18.7)	0.999
ACE-I & ARB	11	(2.1)	3.0 (2.1)	6.0 (3.4)	1.0 (0.7)	0.290
ACE-I or ARB	196	(37.5)	61.0 (43.0)	73.0 (41.5)	46.0 (33.1)	0.187
Aspirin	169	(32.4)	34.0 (23.9)	59.0 (33.5)	63.0 (45.3)	0.001
Beta- blocker	147	(28.2)	36.0 (25.4)	52.0 (29.5)	47.0 (33.8)	0.297
Calcium blocker	152	(29.1)	32.0 (22.5)	52.0 (29.5)	50.0 (36.0)	0.045
Diuretic	121	(23.2)	17.0 (12.0)	37.0 (21.0)	54.0 (38.8)	<0.001
Statin	169	(32.4)	36.0 (25.4)	64.0 (36.4)	57.0 (41.0)	0.016
Vasodilator	18	(3.4)	2.0 (1.4)	7.0 (4.0)	8.0 (5.8)	0.134
**Gout-specific medications (%)**						
*Urate lowering therapies*	53	(10.2)	5.0 (3.5)	17.0 (9.7)	24.0 (17.3)	<0.001
Allopurinol	49	(9.4)	4.0 (2.8)	15.0 (8.5)	23.0 (16.5)	0.001
Febuxostat	4	(0.8)	1.0 (0.7)	2.0 (1.1)	1.0 (0.7)	0.999
*Acute Flare /flare prophylaxis*						
Colchicine	4	(0.8)	0.0 (0.0)	2.0 (1.1)	0.0 (0.0)	0.340
Corticosteroid	21	(4.0)	5.0 (3.5)	11.0 (6.2)	2.0 (1.4)	0.093
NSAIDs	4	(0.8)	2.0 (1.4)	1.0 (0.6)	0.0 (0.0)	0.507

PKD, polycystic kidney disease; BP, blood pressure; LDL, low-density lipoprotein; HDL, high-density lipoprotein; ACE-I, angiotensin-converting enzyme inhibitor; ARB, angiotensin receptor blocker; NSAID, non-steroidal anti-inflammatory medication

### Prevalence of gout

**Fig 1:**
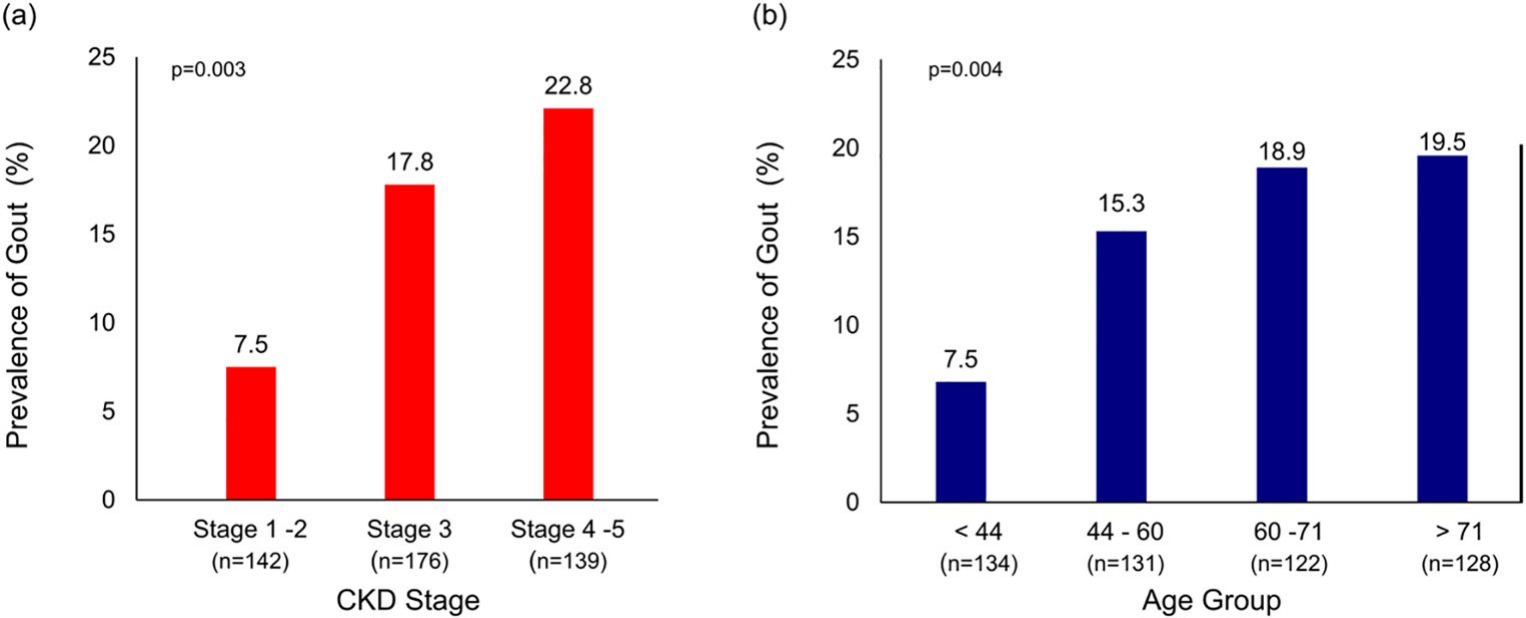
Prevalence of gout by CKD stage and age group. (a) Prevalence of gout by CKD stage groups (b) Prevalence of gout by age group; a proportional trend test was used to assess the linear trend in the proportion of gout cases across age categories.

The overall prevalence of gout in the entire cohort was 16.6% and increased significantly from 7.5% in CKD stages 1–2 to 22.8% in CKD stages 4–5, p < 0.005 (Fig 1). When gout was defined solely by a medical record diagnosis, the prevalence of gout and by CKD stage followed a similar trend (S1 Appendix). The baseline characteristics of the study population by presence or absence of gout are shown in Table 2. Patients with gout were significantly older, predominantly men, and had significantly higher prevalence of coronary disease than those without gout (p<0.005). Overall frequency of gout increased with advancing age; from 7.5% in patients age < 44 years to 19.5% for patients age > 71 years, p<0.005. Compared to patients without gout, those with gout had significantly lower eGFR values [36.3 (19.8) versus 48.1 (26.9) ml/min/1.73m^2^ (p < 0.005) and significantly greater proteinuria [protein/creatinine ratio 323.4 (571) mg/mmol versus 116.1 (174) respectively, all p< 0.01]. Gout patients also experienced significantly lower serum albumin concentrations and significantly higher parathyroid hormone concentrations than those without gout, all p<0.001.

**Table 2:** Baseline characteristics of the study population by presence or absence of Gout.

Variable	n	Overall Cohort (n = 522)	No Gout (n = 393)	Gout (n = 78)	P-value
**Demographic factors**					
Age mean (SD)	515	58.2(16.9)	58.8(16.6)	63.9(13.6)	0.021
Sex					
Men (%)	283	(55.2)	214 (54.7)	54 (69.2)	
Women (%)	230	(44.8)	177 (45.3)	24 (30.8)	0.024
**Race (%)**					
White Irish	479	(95.0)	368 (95.8)	74 (96.1)	0.756
White Irish traveller	2	(0.4)	2 (0.5)	0 (0.0)	0.761
White other	12	(2.4)	5 (1.3)	3 (3.9)	0.756
Asian	2	(0.4)	2 (0.5)	0 (0.0)	0.76
Other	9	(1.8)	7 (1.8)	0 (0.0)	0.755
**Cause of CKD (%)**					
Hypertension	138	(26.8)	117 (29.8)	18 (23.1)	0.273
Diabetes	62	(12.0)	49 (12.5)	13 (16.7)	0.358
Glomerulonephritis	93	(18.1)	66 (16.8)	17 (21.8)	0.328
Autosomal dominant PKD	33	(6.4)	26 (6.6)	6 (7.7)	0.805
Hereditary nephritis	13	(2.5)	12 (3.1)	1 (1.3)	0.704
Other cause of CKD	168	(32.6)	129 (32.8)	22 (28.2)	0.507
Not known	70	(13.6)	49 (12.5)	12 (15.4)	0.464
**Biopsy (%)**					
Native kidney biopsy	75	(14.4)	60 (15.3)	9 (11.5)	0.485
**Comorbid Conditions (%)**					
Diabetes	106	(22.6)	87 (22.2)	19 (24.7)	0.656
Hypertension	370	(78.7)	306 (78.1)	63 (81.8)	0.544
Cancer	33	(7.0)	27 (6.9)	6 (7.8)	0.807
Heart failure	22	(4.7)	19 (4.9)	3 (3.9)	0.999
Thyroid disease	48	(10.2)	42 (10.7)	6 (7.8)	0.54
Stroke or Transient ischaemic attack	22	(4.7)	21 (5.4)	1 (1.3)	0.149
Chronic obstructive airways disease	22	(4.7)	18 (4.6)	4 (5.2)	0.77
Peripheral vascular disease	38	(8.1)	32 (8.2)	6 (7.8)	0.999
Coronary heart disease	75	(16.0)	52 (13.3)	22 (28.6)	0.002
Obesity	28	(6.0)	22 (5.6)	6 (7.8)	0.435
Hypercholesterolaemia	127	(27.1)	106 (27.1)	21 (27.3)	0.999
Depression	20	(4.3)	20 (5.1)	0 (0.0)	0.057
Arthritis	27	(5.7)	23 (5.9)	4 (5.2)	0.999
Osteoporosis	30	(6.4)	25 (6.4)	5 (6.5)	0.999
Current or ex-smoker	52	(11.1)	44 (11.2)	8 (10.4)	0.999
Obesity	28	(6.0)	22 (5.6)	6 (7.8)	0.435
**Physical Measurements**					
Weight (kg)	397	80.7(17.5)	80.1(16.0)	86.7(23.1)	0.094
Pulse (beats/min)	293	75.2(16.3)	74.7(16.2)	76.8(18.2)	0.569
Systolic BP (mmHg)	481	137.7(19.6)	137.8(18.8)	140.2(22.5)	0.576
Diastolic BP (mmHg)	481	77.7(13.3)	77.6(13.7)	78.6(12.1)	0.826
**Laboratory Measures**					
eGFR (mL/min/1.73m^2^)	457	48.4 (27.7)	48.1 (26.9)	36.3 (19.8)	0.001
Haemoglobin (g/dL)	448	12.6 (1.9)	12.6 (1.9)	12.5 (1.8)	0.289
Ferritin (ng/L)	157	253.1 (339.4)	268.6 (361.3)	205.8 (194.9)	0.799
TSAT ratio (%)	126	26.7 (13.4)	27.6 (13.9)	22.9 (8.5)	0.184
Folate (nmol/L)	95	40.2 (143.7)	38.1 (137.8)	58.6 (190.4)	0.215
Vitamin B12 (nmol/L)	119	460.1 (272.1)	471.4 (287.1)	451.2 (160.2)	0.617
Corrected Calcium (mmol/L)	144	2.3 (0.2)	2.3 (0.1)	2.3 (0.3)	0.528
Calcium (mmol/L)	405	2.4 (0.2)	2.4 (0.2)	2.3 (0.1)	0.701
Albumin (g/L)	354	41.1 (5.6)	41.4 (5.8)	38.6 (4.6)	<0.001
Phosphate (mmol/L)	376	1.1 (0.3)	1.1 (0.3)	1.2 (0.3)	0.068
Parathyroid hormone (PTH) (pg/mL)	162	130.6(129.1)	109.8 (95.1)	230.3 (199.6)	<0.001
Total cholesterol (mmol/L)	192	4.6 (1.4)	4.6 (1.4)	4.5 (1.1)	0.783
LDL cholesterol (mmol/L)	124	2.6(1.2)	2.6(1.2)	2.1(0.6)	0.106
HDL cholesterol (mmol/L)	126	1.4(0.5)	1.4(0.5)	1.3(0.4)	0.728
Triglycerides (mmol/L)	190	4.0(21.6)	3.1(17.3)	2.6(1.7)	0.005
HbA1c (mmol/mol)	79	51.6(21.1)	52.5(22.0)	52.5(17.8)	0.850
**Urine tests**					
Protein: creatinine ratio	136	136.0(246.8)	116.1(173.8)	323.4(571.3)	0.009
Albumin: creatinine ratio	51	101.9(214.9)	116.8(233.9)	38.4(33.2)	0.999
**Medications (%)**					
ACE-I	114	(21.8)	93 (23.7)	17 (21.8)	0.772
ARB	93	(17.8)	75 (19.1)	15 (19.2)	0.999
ACE-I & ARB	11	(2.1)	9 (2.3)	2 (2.6)	0.999
ACE-I or ARB	196	(37.5)	159 (40.5)	30 (38.5)	0.801
Aspirin	169	(32.4)	133 (33.8)	31 (39.7)	0.363
Alpha Blocker	18	(3.4)	17 (4.3)	0 (0.0)	0.089
Beta Blocker	147	(28.2)	108 (27.5)	34 (43.6)	0.007
Calcium blocker	152	(29.1)	126 (32.1)	21 (26.9)	0.423
Diuretic	121	(23.2)	84 (21.4)	35 (44.9)	<0.001
Statin	169	(32.4)	140 (35.6)	26 (33.3)	0.795
**Gout-specific Medications (%)**					
*Urate-lowering therapies*	53	(10.2)	0 (0.0)	53 (67.9)	<0.001
Allopurinol	49	(9.4)	0 (0.0)	49 (62.8)	<0.001
Febuxostat	4	(0.8)	0 (0.0)	4 (5.1)	0.001
Probenecid	0	0	0	0	NA
*Acute flare/flare prophylaxis*					
Colchicine	4	(0.8)	0 (0.0)	4 (5.1)	0.001
Corticosteroid	21	(4.0)	15 (3.8)	6 (7.7)	0.136
NSAIDs	4	(0.8)	3 (0.8)	0 (0.0)	0.999

PKD, polycystic kidney disease; BP, blood pressure; LDL, low-density lipoprotein; HDL, high-density lipoprotein; ACE-I, angiotensin-converting enzyme inhibitor; ARB, angiotensin receptor blocker: NSAIDs, Nonsteroidal anti-inflammatory drugs; eGFR, estimated glomerular filtration rate was calculated using the Chronic Kidney Disease Epidemiology Collaboration (CKD-EPI) equation.

### Gout-specific medications

Overall, 67.9% of patients with gout were receiving ULT as shown in Table 2 and the prevalence of ULT use increased from 55.6% in Stages 1–2 to 58.6 % in Stage 3 and to 77.4 % in Stages 4–5 (p = 0.002). Allopurinol was the most commonly prescribed ULT (62.8%) with a much smaller percentage treated with Febuxostat (5.1%). Colchicine use was recorded in 5.1% of patients while a further 7.7% of gout patients were receiving corticosteroids. None of the patients with gout were recorded as using non-steroidal anti-inflammatory drugs (NSAIDS).

### Correlates of gout

The relationship of demographic and clinical factors with gout is illustrated in Table 3. With adjustment only for age and sex; advancing age [1.11 (95% CI 1.02–1.20) for every 5-year increase], male gender [OR 1.85 (1.09–3.12)] and patients with coronary disease [OR 1.94 (1.05–3.58)] were significantly more likely to have gout. Worsening kidney function increased the likelihood of having gout [OR 1.10 (1.03–1.17) for each 5 ml/min/1.73m^2^ decrease in eGFR]. Similarly, higher levels of proteinuria quantified by protein/creatinine ratio estimation were correlated with greater likelihood of gout [log_2_ PCR 1.45 (1.09–1.93), p<0.05). Lower serum albumin levels and worsening secondary hyperparathyroidism were also associated with greater likelihood of gout [OR 1.09 (1.03–1.14) per 1 g/dL decrease in albumin, and OR 1.33 (1.14–1.56) per 50 pg/mL increase in PTH, all p <0.005. Use of diuretics and beta-blockers also correlated with gout [OR 2.72 (1.61–4.60) and OR 1.81 (1.08–3.02) respectively.

**Table 3:** Unadjusted, and age- and sex-adjusted Odd Ratios and 95% confidence intervals for gout.

Variable	N	OR (95% CI)	P-value	N	AOR (95% CI)^1^	P-value
**Demographics**						
Age per 5 year increase	471	1.11 (1.02–1.2)	0.012	469	1.11 (1.02–1.2)	0.013
Male vs Female	469	1.86 (1.11–3.13)	0.019	469	1.85 (1.09–3.12)	0.022
**Comorbid Conditions**						
Diabetes	469	1.15 (0.65–2.03)	0.634	467	0.95 (0.52–1.71)	0.851
Hypertension	469	1.26 (0.68–2.37)	0.463	467	1.14 (0.6–2.16)	0.682
Cancer	469	1.14 (0.46–2.87)	0.777	467	0.89 (0.35–2.29)	0.813
Heart failure	468	0.79 (0.23–2.75)	0.716	466	0.66 (0.19–2.32)	0.519
Thyroid disease	469	0.70 (0.29–1.72)	0.441	467	0.67 (0.27–1.67)	0.388
Stroke or Transient ischaemic attack	469	0.23 (0.03–1.75)	0.157	467	0.21 (0.03–1.57)	0.127
Chronic obstructive airways disease	469	1.14 (0.37–3.46)	0.819	467	1.07 (0.35–3.3)	0.910
Peripheral vascular disease	469	0.95 (0.38–2.36)	0.913	467	0.69 (0.27–1.75)	0.438
Coronary heart disease	468	2.61 (1.47–4.63)	0.001	466	1.94 (1.05–3.58)	0.034
Obesity	469	1.42 (0.56–3.63)	0.462	467	1.21 (0.47–3.13)	0.694
Hypercholesterolaemia	468	1.01 (0.58–1.75)	0.977	466	0.90 (0.52–1.58)	0.725
Depression	469	0.88 (0.3–2.62)	0.817	467	0.80 (0.26–2.42)	0.690
Arthritis	469	1.02 (0.38–2.75)	0.970	467	0.97 (0.35–2.68)	0.960
Osteoporosis	469	0.92 (0.41–2.03)	0.831	467	0.95 (0.42–2.12)	0.892
**Laboratory Variables**						
eGFR per 5 ml/min/1.73m^2^ decrease	419	1.11 (1.04–1.18)	0.001	419	1.10 (1.03–1.17)	0.005
Haemoglobin per 1 g/dL increase	413	0.96 (0.83–1.1)	0.530	411	0.98 (0.85–1.14)	0.838
Ferritin per 1 ng/L increase	151	1.00 (1.00–1.00)	0.450	151	1.00 (1.00–1.00)	0.520
TSAT ratio per 1% increase	121	0.97 (0.92–1.01)	0.167	121	0.97 (0.93–1.02)	0.271
Folate per 1 nmol/L increase	92	1.00 (1.00–1.00)	0.631	92	1.00 (1.00–1.00)	0.499
Vitamin B12 per 1 nmol/L increase	114	1.00 (1.00–1.00)	0.796	114	1.00 (1.00–1.00)	0.892
Calcium (mmol/L)	373	0.56 (0.1–3.04)	0.504	371	0.83 (0.14–4.73)	0.830
Serum Albumin per 1 g/L decrease	327	1.08 (1.03–1.13)	0.002	325	1.09 (1.03–1.14)	0.001
Phosphate per 1 mmol/L increase	346	2.57 (0.97–6.83)	0.059	344	2.43 (0.87–6.81)	0.091
PTH per 50 pg/mL increase	157	1.34 (1.15–1.57)	<0.001	156	1.33 (1.14–1.56)	<0.001
Total cholesterol per 1 mmol/L increase	181	0.92 (0.64–1.32)	0.652	180	1.01 (0.69–1.47)	0.976
LDL cholesterol per 1 mmol/L increase	118	0.55 (0.27–1.15)	0.112	117	0.55 (0.26–1.15)	0.114
HDL cholesterol per 1mmol/L increase	120	0.63 (0.17–2.38)	0.495	119	0.65 (0.16–2.66)	0.545
Triglycerides per 1 mmol/L increase	179	1.00 (0.96–1.03)	0.888	178	1.00 (0.96–1.03)	0.837
HbA1c per 1 mmol/mol increase	75	1.00 (0.97–1.03)	0.993	75	1.00 (0.97–1.03)	0.961
log_2_(PCR)	125	1.43 (1.08–1.9)	0.012	124	1.45 (1.09–1.93)	0.011
**Medications**						
ACE-I	471	0.90 (0.50–1.61)	0.722	469	0.87 (0.48–1.6)	0.665
ARB	471	1.01 (0.54–1.87)	0.976	469	1.19 (0.63–2.25)	0.594
Aspirin	471	1.29 (0.78–2.12)	0.318	469	0.94 (0.55–1.61)	0.829
Beta Blocker	471	2.04 (1.24–3.36)	0.005	469	1.81 (1.08–3.02)	0.023
Calcium blocker	471	0.78 (0.45–1.34)	0.372	469	0.72 (0.41–1.25)	0.243
Diuretic	471	2.99 (1.80–4.97)	<0.001	469	2.72 (1.61–4.60)	<0.001
Statin	471	0.90 (0.54–1.51)	0.699	469	0.76 (0.45–1.29)	0.306

LDL, low-density lipoprotein; HDL, high-density lipoprotein; ACE-I, angiotensin-converting enzyme inhibitor; ARB, angiotensin receptor blocker: NSAIDs, Nonsteroidal anti-inflammatory drugs; eGFR, estimated glomerular filtration rate was calculated using the Chronic Kidney Disease Epidemiology Collaboration (CKD-EPI).

^1^AOR: adjusted for age and sex only

In the fully adjusted model, lower eGFR and worsening serum albumin remained significant correlates of gout as illustrated in Table 4. Given that PTH values were available for a smaller percentage of patients (n = 162), an additional analysis was performed in which we restricted to the final model to those with valid PTH concentrations. In this restricted model, a rising PTH concentration was also significant [OR 1.38, (1.08–1.77) per 50 pg/mL increase, p = 0.01. This model had a C-statistic of 0.83.

**Table 4:** Multivariable Odds Ratio for gout among patients with CKD in the Irish health system.

Variable	AOR (95% CI)	P-value	AOR (95% CI)	P-value
	Model 1 (N = 286)		Model 2 (N = 126)	
Age per 5 year increase	1.09 (0.96–1.24)	0.185	1.00 (0.82–1.21)	0.962
Male vs Female	1.95 (0.95–4.03)	0.070	2.99 (0.79–11.33)	0.107
Coronary Heart Disease	1.57 (0.68–3.63)	0.291	1.47 (0.37–5.76)	0.583
Diuretic use	1.79 (0.87–3.68)	0.111	0.78 (0.20–2.95)	0.710
eGFR per 5 ml/min/1/73m^2^ decrease	1.11 (1.01–1.22)	0.037	1.03 (0.85–1.23)	0.792
Serum albumin per 1 g/L decrease	1.09 (1.02–1.16)	0.008	1.15 (1.01–1.30)	0.040
Serum phosphate per 1 mmol/l increase	0.34 (0.08–1.46)	0.147	0.40 (0.04–4.20)	0.445
Parathyroid hormone per 50 pg/mL increase		–	1.38 (1.08–1.77)	0.011

Model 1: adjusted for continuous variables (age, eGFR, serum albumin, serum phosphate) and categorical variables (sex, history of coronary heart disease and diuretic use). The model had a C-statistic 0.77 and there was no evidence of poor fit from the Hosmer and Lemeshow goodness of fit test (p = 0.6). Model 2: adjusted for all variables as Model 1 in addition to serum parathyroid hormone (PTH). This model had a C-statistic of 0.83 and there was no evidence of poor fit from the Hosmer and Lemeshow goodness of fit test (p = 0.9).

## Discussion

In this large multi-centre study, we found a substantial burden of gout among Irish patients with CKD (16.6%) that increased significantly from 7.5% in patients with mild CKD to 22.8% among patients with moderate to severe CKD (eGFR < 30 ml/min/1.73m^2^). Gout was more common in men than in women and rates increased with advancing age. Adjusting for differences in age and sex, we found that patients with lower kidney function, lower serum albumin, and worsening secondary hyperparathyroidism were more likely to have gout. Overall, almost 68% of gout patients with CKD were receiving ULT agents, with allopurinol being the most commonly prescribed ULT. Importantly and encouragingly, we observed a significant trend of increasing ULT use from 55.6% in Stage 1–2 to 77.4% in Stage 4–5, evidence that supports better recognition and treatment of gout in this high-risk population.

Recent studies have identified strong relationships between gout and CKD, which are not surprising given that hyperuricaemia, the principal driver of gout, is an inevitable consequence of worsening kidney function [17]. However, only few have quantified the burden of gout and corresponding rates of ULT treatment among patients with pre-existing CKD. In our study, we describe a substantial burden of gout in CKD patients attending specialist clinics in the Irish health system. Our estimate (16.6%) is somewhat lower than that reported by the German Chronic Kidney Disease study (24.3%), which may reflect underlying differences in prevalence in the general population from respective countries or differences in rates of death among gout patients with CKD [13]. However, the pattern of increase in gout prevalence from early CKD to advanced CKD followed near identical trends in both studies with an approximately 3-fold rise in prevalence. These results suggest that at the very least that gout is an extremely common but treatable comorbidity among CKD patients who are already under clinical surveillance in the health system.

Our study provides novel insights into the range and extent of treatment strategies for patients with gout managed by kidney specialists in the Irish health system. We report overall treatment rates of 68% for ULT among gout patients with CKD, which are identical to those reported by the German CKD cohort (67%). However, unlike Germany, the principal ULT in Ireland were uricostatic (predominantly allopurinol), as no patient was recorded as receiving uricosuric therapy. Encouragingly, we reveal that rates of ULT use have increased from 55.6% among patients in CKD stage 1–2 to 77.4% for patients with more advanced CKD 4–5 suggesting a propensity to greater ULT prescribing. This trend in prescribing patterns may reflect the increasing recognition by kidney specialists in Ireland that gout is eminently treatable or that gout and/or hyperuricaemia are emerging risk factors for CKD progression [7–11]. It is equally noteworthy, that colchicine (5.1%) and corticosteroids (7.7%) were the standard therapies for acute flare or flare prophylaxis with no recorded use of NSAIDs. This new data highlights a positive practice pattern that supports avoidance of nephrotoxic medications and kidney function preservation [15].

Despite the moderately high proportion of CKD patients with gout receiving ULT therapy in this study, a substantial proportion of patients (~32%) nevertheless were untreated. Studies have consistently shown that the treatment of gout is suboptimal in the general population [18]. Prescription rates of ULT vary substantially from as low as 23% (Taiwan), 38% (UK) and 42% (Sweden) to a spectacular 80% in South Korea [19–21]. Low treatment rates among patients with CKD may be partially explained by the presence of absolute and relative contraindications to the medications, as well as a lack of drug efficacy [22]. Additionally, dietary and lifestyle interventions may account for those not undertaking ULT. Dietary modification is typically suggested as part of the initial treatment for patients with gout, although some suggest it should be ancillary to ULT [23]. Prior to the availability of febuxostat, allopurinol was the principal ULT for patients with gout and impaired renal function. However, the risk of allopurinol hypersensitivity syndrome (AHS) is increased in renal impairment and consequently lingering concerns regarding this infrequent but potentially lethal event may contribute to ULT avoidance in CKD patients [24,25].

Prior studies have identified strong relationships between hyperuricaemia, the precursor to gout, and several metabolic markers [26]. In the current study, we observed for the first time an inverse relationship between serum albumin and gout. In the final adjusted model, for each 1 g/L lower serum albumin, the likelihood of gout increased by 9%. Low serum albumin may reflect protein-losing states, poor nutritional status, or systemic inflammation. Prior studies have also identified an independent relationship between serum uric acid and the extent of albuminuria [27–30]. Tseng and colleagues have reported positive increases in urinary albuminuria with increasing serum uric acid concentrations in type 2 diabetes [27] while prospectively designed cohorts have confirmed the predictive impact of hyperuricaemia on albuminuria [28–30]. Serum albumin is a negative acute phase reactant, and thus low serum albumin levels may also reflect ongoing systemic inflammation associated with gout.

We also uncovered a very strong independent association between elevated PTH and the occurrence of gout. This is an intriguing finding, which suggests that progressive secondary hyperparathyroidism serves to increase the likelihood of gout in the setting of CKD. Previous studies have suggested a strong biological influence of PTH on serum uric acid levels possibly mediated by reduced renal excretion of uric acid [31–35]. However, in virtually all of these studies, hyperparathyroidism was primary in nature, and correction of which led to a fall in uric acid concentrations [31,32]. Our study not only extends the observations of previous instigators but for the first time associates secondary hyperparathyroidism of CKD with clinical gout. Indeed in our analysis, a rising serum PTH level was a stronger correlate of gout than worsening eGFR, suggesting a strong pathobiological basis.

Our study is not with limitations. *First*, the design of the study was cross-sectional in nature with reliance on medical records for data extraction. Therefore, we cannot infer causality between explanatory covariates and gout. *Second*, although joint aspiration for monosodium urate crystals remains the gold standard for diagnosis in clinical practice, our study relied on a clinical diagnosis from medical records based on physician-diagnosis and/or use of ULT. Inclusion of ULT medications ensured greater capture of clinical gout that may not have been recorded on the patient’s medical record. *Third*, we lacked data on serum uric acid concentrations, and thus were unable to correlate gout or ULT use with target thresholds. Notwithstanding these limitations, the study was multicentre, with participation from a diverse range of nephrology clinics across Ireland, thus strengthening the generalisability. Although a probability sampling strategy would have been preferred, we argue that the application of consecutive sampling to a homogenous population, attending renal clinics across multiple sites reduces bias in relation to the representativeness of the study population. The large sample included a detailed description of patient characteristics including comorbid conditions, measures of kidney function including proteinuria. Finally, our analysis included a detailed description of current prescribed ULT medications as well as medications for acute flares and flare prophylaxis.

## Conclusion

In conclusion, our findings demonstrate a substantial burden of gout among patients with CKD in the Irish health system that increases with advancing CKD. Male gender, worsening kidney function, malnutrition and secondary hyperparathyroidism were identified as independent correlates. Almost 68% of patients with CKD were receiving ULT that increased to 77% among patients with Stage 4–5 CKD. Patients with both gout and CKD represent a high-risk group for considerable morbidity and mortality, compounded by the individual contribution of each to adverse clinical outcomes. Given the substantial burden of gout in CKD patients, greater awareness, screening and treatment of gout are key to improving patient outcomes in this population. Future studies should examine dosing of ULT among CKD patients and the extent to which treatment targets are achieved in order to prevent the complications of gout and potentially slow the progression of CKD.

## Supporting information

S1 Appendix Sensitivity analysis of the prevalence of gout, with gout defined solely by a medical record diagnosis. (DOC)
