# Supplementary material for: Prevalence and treatment of gout among patients with chronic kidney disease in the Irish health system: A national study
Source: PLoS One. 2019 Jan 25;14(1):e0210487. doi: 10.1371/journal.pone.0210487 (PMC6347136; doi:10.1371/journal.pone.0210487)
Supplement: S1 Appendix — (DOC) [file pone.0210487.s001.doc]

Sensitivity Analysis

Basing the definition of gout on medical record alone, the overall prevalence of gout was 12.3% and increased significantly from 5.8% in Stage 1-2 CKD to 16.2% in stage 4-5 CKD, P< 0.015. Prevalence varied with age and plateaued at older age groups (5.22%, 13.74%, 13.11%, 13.28% for age groups <44, 44-60, 60-71,>71 years, P = 0.055) and was higher in men than women (14.49 versus 7.39 % P< 0.005). Overall, 56.9% of gout patients with CKD were treated with ULT, and the percentage increased with advancing stage of CKD from 42.9% in Stage 1-2 to 68.2% in Stage 4-5, P<0.005.
